# Vertical transmission of fungal endophytes is widespread in forbs

**DOI:** 10.1002/ece3.953

**Published:** 2014-03-11

**Authors:** Susan Hodgson, Catherine Cates, Joshua Hodgson, Neil J Morley, Brian C Sutton, Alan C Gange

**Affiliations:** School of Biological Sciences, Royal Holloway University of LondonEgham, Surrey, TW20 0EX, U.K

**Keywords:** Fungi, mutualism, plant–plant interactions, pollen, seed, seedling, vertical transmission

## Abstract

To date, it has been thought that endophytic fungi in forbs infect the leaves of their hosts most commonly by air-borne spores (termed “horizontal transmission”). Here, we show that vertical transmission from mother plant to offspring, via seeds, occurs in six forb species (*Centaurea cyanus, C. nigra*,*Papaver rhoeas*,*Plantago lanceolata*,*Rumex acetosa,* and *Senecio vulgaris*), suggesting that this may be a widespread phenomenon. Mature seeds were collected from field-grown plants and endophytes isolated from these, and from subsequent cotyledons and true leaves of seedlings, grown in sterile conditions. Most seeds contain one species of fungus, although the identity of the endophyte differs between plant species. Strong evidence for vertical transmission was found for two endophyte species, *Alternaria alternata* and *Cladosporium sphaerospermum*. These fungi were recovered from within seeds, cotyledons, and true leaves, although the plant species they were associated with differed. Vertical transmission appears to be an imperfect process, and germination seems to present a bottleneck for fungal growth. We also found that *A. alternata* and *C. sphaerospermum* occur on, and within pollen grains, showing that endophyte transmission can be both within and between plant generations. Fungal growth with the pollen tube is likely to be the way in which endophytes enter the developing seed. The fact that true vertical transmission seems common suggests a more mutualistic association between these fungi and their hosts than has previously been thought, and possession of endophytes by seedling plants could have far-reaching ecological consequences. Seedlings may have different growth rates and be better protected against herbivores and pathogens, dependent on the fungi that were present in the mother plant. This would represent a novel case of trans-generational resistance in plants.

## Introduction

Every living plant hosts a diverse array of fungi and bacteria that reside on the exterior surface or inhabit the interior of the tissues. Within the latter group, those microbes that cause no visible signs of infection are referred to as “endophytes” and consist of a vast array of species, with different life histories (Rodriguez et al. 2009). Fungal endophytes are well studied in the Graminae, because their ability to confer herbivore resistance renders them important from the ecological and economic point of view. Certain grass endophytes, such as *Neotyphodium coenophialum* (Morgan-Jones et Gams) Glenn, Bacon et Hanlin, have come to be regarded as classic examples of mutualism; the endophyte never leaves its host, being vertically transmitted from parent to offspring via seeds, while its presence renders host tissues toxic to both mammalian and insect herbivores (Saikkonen et al. 2010). These fungi are often referred to as “true endophytes.”

Appealing although the mutualism argument may be, it is actually based on a relatively small collection of studies involving a few grass species and associated herbivores (Saikkonen et al. 2010). Furthermore, the transmission process is far from perfect, and the endophyte can be lost at all growth stages in the life of a plant (Afkhami and Rudgers 2008). Complicating the issue further is the fact that many other endophyte fungi in grasses are transmitted horizontally, via soil- or air-borne spores (Sánchez Márquez et al. 2012), and the role of these endophytes in their hosts is largely unknown.

Endophytes in forbs (i.e., herbaceous eudicots) appear to form diverse communities (Gange et al. 2007; Wearn et al. 2012) and are also thought to be predominantly horizontally transmitted (Rodriguez et al. 2009). This assumption is primarily based upon the fact that many of the fungi concerned (e.g., species of *Alternaria*,*Cladosporium,* and *Epicoccum*) are ubiquitous saprophytes, sporulating in soil or on dead leaf material (Hayes 1979). It is not an unreasonable assumption, as these endophytes are extremely common components of aerial spore populations (e.g., Marchisio and Airaudi 2001). Indeed, Sánchez Márquez et al. (2012) suggest that these fungi are incapable of vertical transmission, via seeds.

If we extend the grass-endophyte mutualism argument to forbs, then one would not expect these ubiquitous fungi to confer benefits to their hosts, in terms of resistance to pests and pathogens (Saikkonen et al. 2010). The dominant taxa, such as *Alternaria, Cladosporium,* and *Epicoccum,* seem not to be host specific and are opportunistic colonizers of very many plants (Rodriguez et al. 2009). However, there is an accumulating body of evidence that suggests these fungi can enhance the resistance of their hosts to insect herbivores (e.g., McGee 2002; Jaber and Vidal 2010; Gange et al. 2012) and pathogens (Gao et al. 2010). This raises the intriguing question as to whether these fungal endophytes exist in a more mutualistic relationship with their hosts than was previously thought, perhaps being involved in vertical transmission, like *N. coenophialum* in grasses.

To date, vertical transmission of endophytes has been recorded in a few species of forbs, particularly with the fungus *Undifilum oxytropis* in species of locoweeds (*Astragulus* and *Oxytropis sp*.). This fungus appears to inhabit all tissues of the plants (Cook et al. 2009, 2011), and there is good evidence that the endophyte present in seeds is transferred to seedlings, thereby conferring herbivore resistance via alkaloid production (Oldrup et al. 2010; Ralphs et al. 2011). Similar alkaloid production and transmission via seeds by an unidentified fungus has also recently been reported in *Ipomoea carnea* by Cook et al. (2013). However, to date, such vertical transmission has not been observed with ubiquitous endophytes, such as *Alternaria* and *Cladosporium*. The phenomenon is well known with pathogenic fungi (e.g., Oliver et al. 2001; Galperin et al. 2003) and in some pathogens with latent endophytic phases (Sowley et al. 2010). The majority of pathogenic fungal transmission is probably on the exterior of the seed coat, and indeed, countless ecological experiments recognize this fact by describing surface sterilization of seeds as the first step in their methods.

The main aim of our research was to determine whether true vertical fungal transmission is likely to be a widespread phenomenon in ubiquitous endophytes. Our primary hypothesis was that vertical transmission does occur frequently, given that many of the very common endophyte species have been recovered from within seeds of forbs (e.g., D'Amico et al. 2008).

If vertical transmission does occur, then the question arises as to how the endophytes arrive at, and in, the developing seed. There is remarkably little evidence that these fungi exhibit systemic growth within forbs, unlike the true endophytes in grasses. Sánchez Márquez et al. (2012) state that they are incapable of doing so, a statement that appears to be corroborated by infection experiments of Jaber and Vidal (2010) and Gange et al. (2012). Furthermore, reports of endophytes from the floral parts of forbs (petals and stamens) appear to be absent. We therefore developed a second hypothesis that endophytes are transmitted via the pollen. Endophytic bacteria have been reported from pollen (Madmony et al. 2005), and Marr (1998) provides photographic evidence of spores of the pathogenic fungus *Microbotryum violaceum* (Pers.) G. Deml & Oberw. attached to pollen grains of *Silene acaulis* (L.) Jacq. Furthermore, fungi that occur as endophytes have been recovered from the pollen load of certain bees (Osintseva and Chekryga 2008). Pollen grains exhibit elaborate and ornate external structures for which a number of functions are suggested (Edlund et al. 2004), but these structures also provide good opportunities for the attachment of fungal spores. The mycology of pollen is extremely poorly known, but such transfer would represent a novel endophyte transmission system within plant generations, perhaps enabling subsequent vertical transmission to occur.

## Materials and Methods

### Plant species studied

Three annual and three perennial plant species were chosen for the study, all of which were growing in close proximity in a mixed grassland community on the campus of Royal Holloway University of London, described in Wearn and Gange (2007) and Wearn et al. (2012). The annual species were *Centaurea cyanus* L. (Asteraceae), *Papaver rhoeas* L. (Papaveraceae), and *Senecio vulgaris* L. (Asteraceae), while the perennial species were *Centaurea nigra* L. (Asteraceae), *Plantago lanceolata* L. (Plantaginaceae), and *Rumex acetosa* L. (Polygonaceae). All plants were sampled in July 2011, when individuals were mature, with a mixture of open flowers and ripe seeds. The meadow is mown annually in late summer, and no chemicals have ever been applied.

### Isolation of endophytes from pollen

Thirty plants of each species were chosen at random from the meadow; all plants were growing within 30 m of each other. Ten stamens were removed at random from each plant, and five were subjected to the method III sterilization procedure of Schulz et al. (1993), while five were left unsterilized. For sterilization, stamens were placed on sterile filter paper and subjected to serial immersion in ethanol, sodium hypochlorite, and sterile water. All pollen from each stamen was transferred with a sterile blade to potato dextrose agar (PDA) containing 80 mg L^−1^ streptomycin sulfate and 60 mg L^−1^ penicillin G added to inhibit bacterial contamination. Plates were incubated in the light at 20°C, and fungal isolates were removed soon after they appeared, to eliminate confusion through overgrowth on a plate. Each isolate was subcultured onto potato carrot agar (PCA) to induce sporulation to aid identification. After a minimum of 8 weeks on PCA, all isolated fungi were identified by B. C. Sutton.

### Isolation of endophytes from seeds and seedlings

Ninety mature seeds from each plant were taken and surface sterilized using the method above, while a further 90 were left unsterilized. These were plated in groups of three on PDA plates, and fungal colonies were isolated as above and subcultured on PCA. In addition, a further 90 seeds were surface sterilized and the testa gently broken in sterile water before plating on to PDA. This was carried out in order to examine whether the intact testa was a barrier to endophyte appearance. All plates were sealed and incubated as above.

In this study, we used the sterile seedling method of Ernst et al. (2003). Thus, a further 100 seeds from each plant species were surface sterilized as above. These were placed in 90-mm-diameter petri dishes containing sterile filter paper, moistened with sterile water. The dishes were sealed with Parafilm® and placed in a constant environment (CE) room at 20°C, with a light regime of 16:8 L:D until germination occurred. Upon germination, 50 seedlings (of identical size and development) of each species were transferred into sterile Falcon™ tubes, containing wet sterile filter paper, to continue their growth and the development of true leaves. Filter papers were moistened with 10 mL of a quarter strength-balanced nutrient solution, prepared with sterile water, to provide sufficient nutrients for seedling growth. When the first true leaf appeared, the cotyledons and the true leaf were removed, and surface sterilized as above. The edges of each were cut with a sterile blade, and the fragments placed on PDA. Fungal colonies were isolated as above and subcultured on PCA for a minimum of 8 weeks.

### Data analysis

All analyses were conducted using plants as replicates. Isolation frequency (IF) of each fungus in each plant part was calculated by dividing the total number of colonies (isolates) of that species by the total number of colonies of all species isolated.

Differences in species richness of endophytes and fungal IF between plant parts were examined with one-way analysis of variance, with all percentage data subjected to the arc sine transformation prior to analysis. All analyses were conducted with the UNISTAT® (London, UK) statistical package.

## Results

A total of 26 different endophytes were found across all plants, but different fungi were recovered from each of the six plant species (Table 1). Two species of fungi (*Alternaria alternata* (Fr.) Keissl. and *Cladosporium sphaerospermum* Penz.) dominated the assemblages and together with *Tricothecium roseum* (Pers.) Link were the only endophytes to be recovered from all six plant species. Total endophyte species per plant also varied, being lowest in *P. rhoeas,* and highest in *S. vulgaris* (Table 1).

**Table 1:** Occurrence of endophytes in each of the six forbs. Values are the proportion of plants (*n *= 30) that contained each fungus

	*Centaurea cyanus*	*Papaver rhoeas*	*Senecio vulgaris*	*Centaurea nigra*	*Plantago lanceolata*	*Rumex acetosa*
*Acremonium strictum*	3.3	0	0	0	3.3	6.7
*Alternaria alternata*	43.3	53.3	100	66.7	93.3	96.7
*Aspergillus niger*	13.3	0	3.3	30	0	26.7
*Aureobasidium pullulans*	0	0	3.3	0	0	0
*Botrytis cinerea*	0	0	0	3.3	0	0
*Chaetomium cochliodes*	6.7	0	13.3	0	3.3	0
*Cladosporium cladosporioides*	13.3	0	23.3	26.7	10	33.3
*Cladosporium oxysporum*	0	0	3.3	3.3	3.3	3.3
*Cladosporium sphaerospermum*	100	100	23.3	100	63.3	76.6
*Colletotrichum coccodes*	13.3	23.3	0	0	0	0
*Colletotrichum dematium*	16.7	0	0	3.3	0	0
*Epicoccum nigrum*	0	3.3	33.3	16.7	13.3	43.3
*Fusarium avenaceum*	0	0	6.7	0	0	0
*Fusarium equiseti*	0	0	13.3	0	0	10
*Fusarium merismoides*	0	0	16.7	0	0	6.7
*Fusarium tricinctum*	0	0	23.3	30	0	3.3
*Fusarium sp. A*	0	0	3.3	0	0	0
*Geotrichum candidum*	0	0	0	0	3.3	0
*Mucor hiemalis*	0	0	3.3	0	6.7	6.7
*Penicillium sp. A*	3.3	0	3.3	0	0	0
*Penicillium sp. B*	3.3	0	0	0	0	0
*Phialophora verrucosa*	0	0	3.3	0	0	33.3
*Rhabdospora coricea*	0	0	3.3	0	0	0
sterile sp. A	3.3	0	0	0	0	0
sterile sp. B	0	0	0	0	16.7	0
*Tricothecium roseum*	13.3	23.3	13.3	6.7	16.7	26.7
Total fungal species per plant species	12	5	18	10	11	13

**Figure 1:**
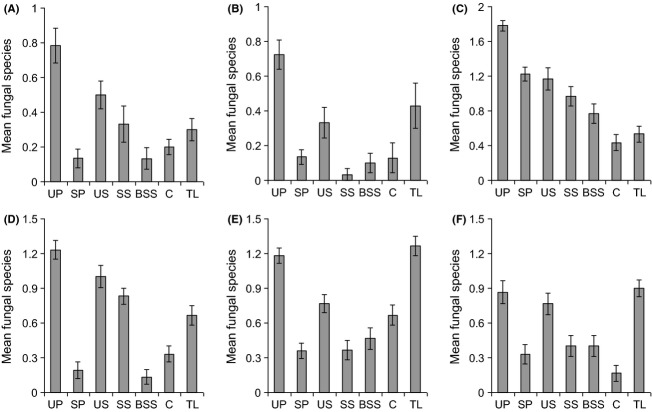
Average number of endophyte fungal species per plant organ (pollen per stamen, seeds, or leaves) in six forbs. (A) *Centaurea cyanus*, (B) *Papaver rhoeas*, (C) *Senecio vulgaris*, (D) *Centaurea nigra*, (E) *Plantago lanceolata*, and (F) *Rumex acetosa*. Key to axis labels: UP, unsterilized pollen; SP, sterilized pollen; US, unsterilized seeds; SS, sterilized seeds; BSS, broken, sterilized seeds; C, cotyledon; TL, true leaves. Vertical lines represent one standard error.

There were significant differences in endophyte species richness isolated from different plant parts (Fig. 1, Table 2), with unsterilized pollen tending to yield the most species. In all plant species, endophytes were isolated from both unsterilized and sterilized pollen, indicating that these fungi occur on and within pollen grains. Unsterilized pollen yielded more endophyte species than sterilized pollen in all plant species (Fig. 1).

**Table 2:** Summary of ANOVA testing for differences in endophyte species richness between plant parts in each of the six plant species studied. All degrees of freedom for these analyses: 6,203. Also tabulated are summaries of tests for differences in infection frequency between plant parts of *Alternaria alternata* and *Cladosporium sphaerospermum*. Degrees of freedom: 6,203, except for *C. sphaerospermum* in *Centaurea cyanus* (4,145) and in *P. rhoeas* and *C. nigra* (5,174)

	*F*	*P*
Species richness		
*Centaurea cyanus*	7.48	<0.001
*Papaver rhoeas*	19.22	<0.001
*Senecio vulgaris*	21.73	<0.001
*Centaurea nigra*	40.88	<0.001
*Plantago lanceolata*	24.18	<0.001
*Rumex acetosa*	12.73	<0.001
Infection frequency		
*Alternaria alternata* in:		
*Senecio vulgaris*	5.40	<0.01
*Plantago lanceolata*	20.82	<0.001
*Rumex acetosa*	15.85	<0.001
*C. sphaerospermum* in:		
*Centaurea cyanus*	1.35	N.S.
*Papaver rhoeas*	4.08	<0.01
*Centaurea nigra*	6.43	<0.001

In general, more endophyte species were recovered from unsterilized than sterilized seeds, while breaking of the testa did not seem to allow greater recovery of endophytes. Indeed, the opposite was true in two plants (*C. cyanus* and *C. nigra*), where more endophytes were found in sterilized seeds compared with sterilized broken seeds (Fig. 1A,D). Generally, considerably more endophyte species were recovered from true leaves than cotyledons, the exceptions being *C. cyanus* and *S. vulgaris,* where numbers were similar (Fig. 1A,C).

**Figure 2:**
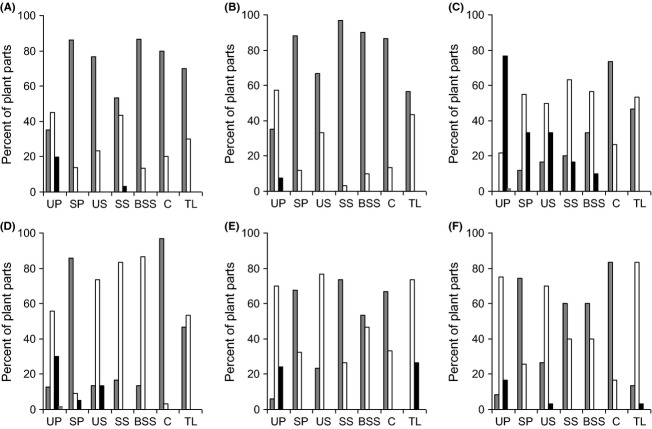
The percentage of plant parts that yielded zero (dark gray bars), one (white bars), two (black bars), or three (pale gray bars) endophyte species. (A) *Centaurea cyanus*, (B) *Papaver rhoeas*, (C) *Senecio vulgaris*, (D) *Centaurea nigra*, (E) *Plantago lanceolata*, (F) *Rumex acetosa*. Key to axis labels as in Fig. 1.

In virtually all plant species, the vast majority of plant parts yielded no or one endophyte (Fig. 2). The single exception to this was *S. vulgaris*, in which 76% of unsterilized pollen samples produced two endophytes (Fig. 2C). Some clear patterns emerged from these frequency data. Between 50 and 75% of unsterilized pollen samples yielded one or two endophytes, while in sterilized pollen, these figures dropped to 30% or less. In most species, the proportion of unsterilized seeds producing one endophyte was higher than that of sterilized seeds, the exceptions being *C. nigra*, where they were similar and *C. cyanus*, in which 43% of sterilized seeds produced an endophyte, compared with only 23% in unsterilized seeds.

Perhaps the clearest pattern was seen in cotyledons and true leaves. In all species, the majority of cotyledons produced no fungi (up to 96% in *C. nigra*), while the majority of true leaves yielded one or two fungi. The most dramatic changes were seen in *C. nigra* where the percent of samples producing one fungus increased from 3.3% in cotyledons to 53% in true leaves (Fig. 2D) and in *R. acetosa*, where the corresponding figures went from 16% to 83% (Fig. 2F).

**Figure 3:**
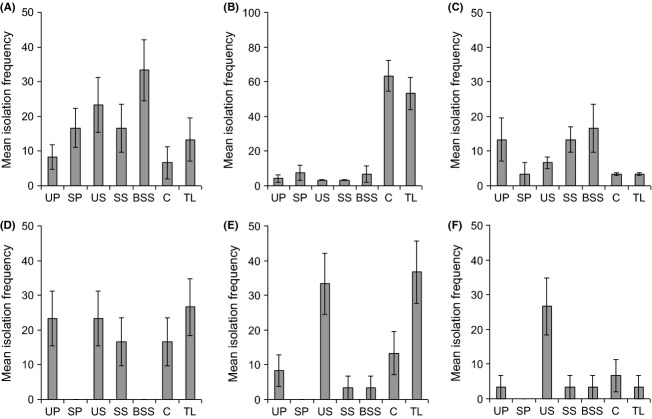
Mean isolation frequency of endophytes common to all, or the majority of, plant parts in each plant species. (A) *Alternaria alternata* in *Senecio vulgaris*; (B) *A. alternata* in *Plantago lanceolata*; (C) *A. alternata* in *Rumex acetosa;* (D) *Cladosporium sphaerospermum* in *Centaurea cyanus*; (E) *C. sphaerospermum* in *Papaver rhoeas*; (F) *C. sphaerospermum* in *C. nigra*. Vertical lines represent one standard error. Key to axis labels as in Fig. 1.

In all plant species, only one fungal species was recovered from all, or virtually all, plant parts. In *P. lanceolata*,*R. acetosa,* and *S. vulgaris*, this was *Alternaria alternata*, while in *C. nigra*,*C. cyanus,* and *P. rhoeas,* it was *Cladosporium sphaerospermum* (Fig. 3). No other fungal species was found in more than two different plant parts. In *S. vulgaris* and *R. acetosa*,*A. alternata* was rarer in leaves than all other structures (Table 2, Fig. 3A,C). However, the opposite was true in *P. lanceolata,* where it occurred at low frequency in pollen and seeds, but showed a remarkable increase in isolation frequency in leaves (Fig. 3B).

*Cladosporium sphaerospermum* showed different patterns of abundance to *A. alternata* and also differed between plant species. It did not differ between plant parts in *C. cyanus*, and in *P. rhoeas,* it was commonest on unsterilized seeds and in true leaves, while in *C. nigra,* it was most frequently recovered from unsterilized seeds (Table 2, Fig. 3D–F).

## Discussion

We report two entirely novel facts about endophytes in forbs, which could have far-reaching consequences for our understanding of the ecological role of these fungi in plants. Firstly, endophytes can be transmitted on and inside pollen grains, and secondly, certain fungi associated with pollen occur also on and in ripe seeds and inside the cotyledons and true leaves of seedlings, when grown in aseptic conditions. This provides a clear demonstration of vertical transmission of these fungi in a range of annual and perennial forbs. Endophytes thus exhibit transfer both within and between generations of plants. The fact that other endophytes in forbs show vertical transmission (e.g., Oldrup et al. 2010; Cook et al. 2013) and the consistency of pattern across all six plant species studied here suggests that this is a widespread phenomenon and not isolated occurrences. Our field sampling was restricted to one site, but there is no evidence that infection levels of the fungi were particularly high in this area, compared with nearby sites (Gange et al. 2007, 2012).

It is possible that had we used molecular techniques, a higher diversity of endophytes would have been found and that some of our zero values are underestimates of diversity. However, the limited evidence suggests that in plants where foliage dies and grows anew each year, the vast majority of endophytes are culturable (Hodgson 2010). Furthermore, subjecting plant tissues to PCR methods followed by T-RFLP produced an identical number of Operational Taxonomic Units (13) in *P. lanceolata* to the number of species identified by culturing (Hodgson 2010). It appears that it is in long-lived woody plant parts where the two methods may produce different results (Arnold 2007). Hence, we believe that the method used here has provided an accurate measure of fungal occurrence in the plant species studied.

Reports of fungi associated with pollen grains on plants are rare, although a few plant pathogens are believed to be transmitted via pollen (Card et al. 2007). The intricacy of the surface of many pollen grains could provide ample opportunity for the attachment of fungal spores or hyphae (Marr 1998; Edlund et al. 2004). However, the fact that some fungal species were recovered from sterilized pollen indicates that the association between endophytes and pollen is considerably more intimate than simple exterior adhesion. While we have not actually shown the movement of fungi from pollen grain to developing ovule, we have shown the potential for transfer of fungi from one individual living plant to another. To date, it has been assumed that these fungi sporulate on senescent plant material, with air-borne spores then infecting living tissues of other plants (Rodriguez et al. 2009). Our results demonstrate a novel form of fungal transfer between living plants, within generations. Given that the same fungal species were found on and within both pollen and seed of each plant species, we suggest that there is a high probability that fungal growth occurs as the pollen tube is formed. This would be analogous to the manner in which plant pathogenic fungal spores can alight on the stigma and grow into the developing fruit (Ngugi and Scherm 2004).

In this study, most unsterilized seeds yielded one or two endophyte species, while sterilized seeds yielded none, or one species. Previous studies of seed endophytes in forbs are limited, but seem to suggest that one endophyte per seed is a common occurrence (Shipunov et al. 2008). Interestingly, breaking of the testa did not allow for the recovery of more fungi, suggesting that the seed coat is not a barrier to fungal growth. However, seed germination does seem to present a bottleneck for fungal transmission, as in four of the six plant species, species richness of endophytes was lower in cotyledons than seeds. This suggests that the transmission process is an imperfect one, as happens with grass endophytes (Afkhami and Rudgers 2008). Meanwhile, in all six plant species, fungal occurrence in true leaves was equal to or greater than that in cotyledons. As seedlings were grown in sterile conditions, this is clear evidence for systemic growth of the fungi within the plant, contrary to the statement of Sánchez Márquez et al. (2012), who state that most ubiquitous endophytes are incapable of such growth. It is exceptionally unlikely that contamination of true leaves occurred, given the large number of replicates in the experiment that yielded no fungi, the consistency of fungal species occurrence and that no exterior fungal growth was observed in the experimental units. These results suggest that fungal systemic growth occurs as the first true leaves are produced.

Two fungi were found in all or nearly all plant parts sampled. In *P. lanceolata*,*R. acetosa,* and *S. vulgaris,* this was *A. alternata*, while in *C. cyanus*,*C. nigra,* and *P. rhoeas,* it was *C. sphaerospermum*. The consistency of fungal occurrence across plant parts, particularly with *A. alternata*, provides very strong evidence for vertical transmission of these fungi in the plants concerned, upholding our original hypothesis. Both of these fungi are common in species lists of endophytes from a range of forbs, but this is the first report of their vertical transmission and occurrence in seedlings of such plants. Because they are so common in aerial spore populations (e.g., Marchisio and Airaudi 2001), it is possible that they were present as contaminants within our system. We believe this to be extremely unlikely for several reasons. Firstly, neither fungus was isolated from every plant part, and both showed differences in infection frequency between different plant parts. If our results reflected random spore contamination, then one would get similar frequencies across plants and plant organs. Secondly, one would not expect to see the observed plant species-specific differences for the same reason. All studies took place in a sterile cabinet, and we believe that our results are genuine and not artefacts of the spore rain. Furthermore, it is interesting that particular species of endophytes seem to be associated with certain plants. Such species specificity has been noted before with these fungi (Gange et al. 2007; Wearn et al. 2012) and might not be expected from their almost ubiquitous distribution in nature (Hayes 1979). Therefore, these results combined with their known plant protective effects (Gange et al. 2012) suggest that endophytes of forbs may exist in more mutualistic associations with their hosts than has previously been thought (Rodriguez et al. 2009).

The fact that some seedlings contain endophytes and others do not could have far-reaching consequences for plant population dynamics. Indeed, the vertically transmitted endophyte *Undifilum oxytropis* can confer high levels of alkaloid content in the tissues of some seeds and seedlings of locoweeds (Ralphs et al. 2011; Grum et al. 2012). Of the fungi studied here, *Alternaria alternata* may have negative effects on insect herbivores (Abbas and Mulrooney 1994) and on plant pathogenic fungi (Musetti et al. 2006) or be negatively associated with other endophytes (Gange et al. 2007). Some strains can also cause disease in plants, although we saw no evidence of this in our sterile seedlings. Meanwhile, *C. sphaerospermum* can produce gibberellins and so promote plant growth (Hamayun et al. 2009). Furthermore, endophytes in tropical tree seedlings can protect those young plants against foliar pathogens (Arnold et al. 2003). These effects, taken together with the fact that endophyte occurrence is not universal or evenly distributed across seedlings, will result in a wide variation in potential seedling growth rates. Even in the absence of competition, plant size distributions mostly conform to the log-normal, in which there are many small and a few large individuals. Traditionally, it has long been thought that variation in seed size and seedling growth rate generated such size hierarchies (Waller 1985). However, our data suggest that endophytes should also be considered as a possible cause. Such variation will have important consequences for individual life histories, as a larger seedling may produce a larger plant that in turn may be more attractive to insect herbivores (Schoonhoven et al. 2005). Moreover, recent studies suggest that trans-generational resistance occurs in plants, whereby mother plants can pass signatures of attack to the next generation, thereby altering the resistance to herbivores of their seedlings (Rasmann et al. 2012). Our data suggest a novel mechanism of trans-generational resistance, mediated by endophyte fungi. Mother plants that possess certain fungi can be better defended against insects (Gange et al. 2012) and be larger (Jaber and Vidal 2009) and so produce more offspring. We have shown that their endophytes can be passed to some of these offspring, and this may mean altered and variable resistance of seedling plants to herbivores and pathogens. This intriguing idea is the subject of our current research.

In grasses, vertically transmitted endophytes have been considered as a good example of a mutualism (Saikkonen et al. 2010). However, to date, there has existed a paradox in endophyte ecology in forbs. Traditionally, it has been thought that these ubiquitous fungi are horizontally transmitted; thus, their relationship with the plant should be considerably looser, and we might not expect to see examples of mutualistic interactions (Rodriguez et al. 2009). However, there is an accumulating body of evidence that shows protective effects of the endophytes on their hosts (e.g., McGee 2002; Jaber and Vidal 2010; Gange et al. 2012). We believe there is no longer a paradox; endophytes do exhibit widespread vertical transmission in forbs. In so doing, they may exist in much tighter relations with their hosts than has been thought, and some of them may be mutualistic, in a similar manner to the “true” endophytes in grasses. The concept of mutualism with these endophytes is an intriguing one which is not shown by our study, but it is certainly one that would merit further study.
